# RNAP II produces capped 18S and 25S ribosomal RNAs resistant to 5′-monophosphate dependent processive 5′ to 3′ exonuclease in polymerase switched *Saccharomyces cerevisiae*

**DOI:** 10.1186/s12860-022-00417-6

**Published:** 2022-04-10

**Authors:** Miguel A. Rocha, Bhavani S. Gowda, Jacob Fleischmann

**Affiliations:** 1 Research Division, Greater Los Angeles VA Healthcare System, Los Angeles, California USA; 2 David Geffen School of Medicine at UCLA, Los Angeles, California USA; 3 School of Dentistry at UCLA, Los Angeles, California USA

## Abstract

**Background:**

We have previously found that, in the pathogenic yeast *Candida albicans,* 18S and 25S ribosomal RNA components, containing more than one phosphate on their 5′-end were resistant to 5′-monophosphate requiring 5′ → 3″ exonuclease. Several lines of evidence pointed to RNAP II as the enzyme producing them.

**Results:**

We now show the production of such 18S and 25S rRNAs in *Saccharomyces cerevisiae* that have been permanently switched to RNAP II (due to deletion of part of RNAP I upstream activator alone, or in combination with deletion of one component of RNAP I itself). They contain more than one phosphate at their 5′-end and an anti-cap specific antibody binds to them indicating capping of these molecules. These molecules are found in RNA isolated from nuclei, therefore are unlikely to have been modified in the cytoplasm.

**Conclusions:**

Our data confirm the existence of such molecules and firmly establish RNAP II playing a role in their production. The fact that we see these molecules in wild type *Saccharomyces cerevisiae* indicates that they are not only a result of mutations but are part of the cells physiology. This adds another way RNAP II is involved in ribosome production in addition to their role in the production of ribosome associated proteins.

**Supplementary Information:**

The online version contains supplementary material available at 10.1186/s12860-022-00417-6.

## Background

Eukaryotic cells devote a large percentage of their energy resources to the production of ribosomes [1], the protein producing organelles located in the cytoplasm. They are made up of structural and synthetically active RNAs combined with over 70 proteins [2]. In yeast, the generation details of the rRNA components are well established. The genes coding for rRNAs are grouped in tandem repeats separated by non-transcribed spacer sequences (NTS) [3]. The NTS contains the rDNA promoter with its upstream element (UE) and core element (CE) representing the initiation site of rDNA transcription [4]. This transcription requires the binding of upstream activating factor (UAF), a multiprotein complex consisting of Rrn5, Rrn9, Rrn10, Uaf30, histones H3 and H4, to the upstream element and TATA binding protein (TBP) [5, 6]. The transcription of rDNA is carried out by RNA polymerase I (RNAP I) resulting in a 35S rRNA precursor molecule processed into 18S, 25S and 5.8S rRNA components [7, 8]. The gene for the fourth component 5S, is located within the NTS, and transcribed by RNA polymerase III (RNAP III) in the reverse direction [9]. These components are mostly assembled with the ribosomal proteins in the nucleus and are exported and completed in the cytoplasm [10]. The genes coding for the ribosomal proteins are transcribed by RNA polymerase II (RNAP II) thus giving all three RNA polymerases a role in ribosome biogenesis [11].

Since ribosomal RNA (rRNA) represents over 80% of total RNA produced by cells, a major function for rRNA has become to serve as control for quality and quantity of RNA isolation, for studies that focus on the many coding and non-coding smaller RNAs [12]. 5′-monophosphate dependent 5′ to 3′ processive exonucleases, such as Terminator (Lucigen), have become available, to enhance the recovery of RNAs of interest, by eliminating the dominating rRNA from total isolated RNA. The utility of these enzymes is based on the fact that processed RNA molecules typically have a single phosphate on their 5′-end, making them vulnerable to these enzymes [13]. Capped RNAs typically are protected from digestion, aiding in their recovery. In studies utilizing Terminator involving the polymorphic yeast *Candida albicans,* we unexpectedly found that this yeast was producing 18S and 25S rRNA components resistant to digestion as it shifted to the stationary growth phase [14]. Digestion of rRNA with tobacco acid pyrophosphatase made these 18S and 25S molecules again susceptible to Terminator digestion. This indicated that the 5′-ends of these Terminator resistant molecules, contained more than one phosphate. Additional studies with the same yeast, that included RNAP I inhibition, chromatin immune precipitation with anti-RNAP II antibodies and immunoblotting with anti-cap specific antibodies were carried out [15]. The sum of these experiments indicated that in addition to its role in ribosomal protein production, RNAP II may play a role in the production of these exonuclease resistant RNA molecules.

While typically RNAP II is suppressed from gaining access to the rDNA promoter site, allowing RNAP I an exclusive role in rRNA transcription, in *Saccharomyces cerevisiae* a role for RNAP II in rRNA production is well established [16, 17]. This yeast contains tandem repeats of rDNAs of 9.1-kb length on chromosome XII as many as 200, but can also have a 9.1-kb monomer episomal circles of rDNA [18]. They are excisional products of homologous recombination between tandem repeats. Such an episomal circular rDNA containing respiratory deficient *Saccharomyces cerevisiae* with a cryptic RNAP II promoter has been described [19]. This organism could utilize RNAP II to generate a 35S precursor off the episomal rDNA circle, while possibly utilizing RNAP I for copying off the tandem repeats. Similarly, mutants with *UAF30* deletion also utilize both RNAP I and RNAP II for rRNA transcription [6]. Deletion of *rrn9*, one of the UAF components results in complete switching to RNA II, designated as a PSW phenotype [20]. Additional deletion of RPA 135 component of RNAP I in these PSW yeast, confirmed the sole role of RNAP II in rRNA transcription in these cells [21, 22]. Primer extension studies of the 5′-end of the polycistronic 35S precursor molecules showed them to be variable from − 9 to − 95 from the known RNAP I promoter site, suggesting a separate or overlapping promoter for RNAP II in these mutants. Recently, we became aware of the availability of such PSW phenotypes of *Saccharomyces cerevisiae* from YGRC/NBRP in Japan, which allowed us to test the validity of RNAP II’s role in the production of exonuclease resistant 18S and 25S rRNA components.

## Results

**Fig. 1:**
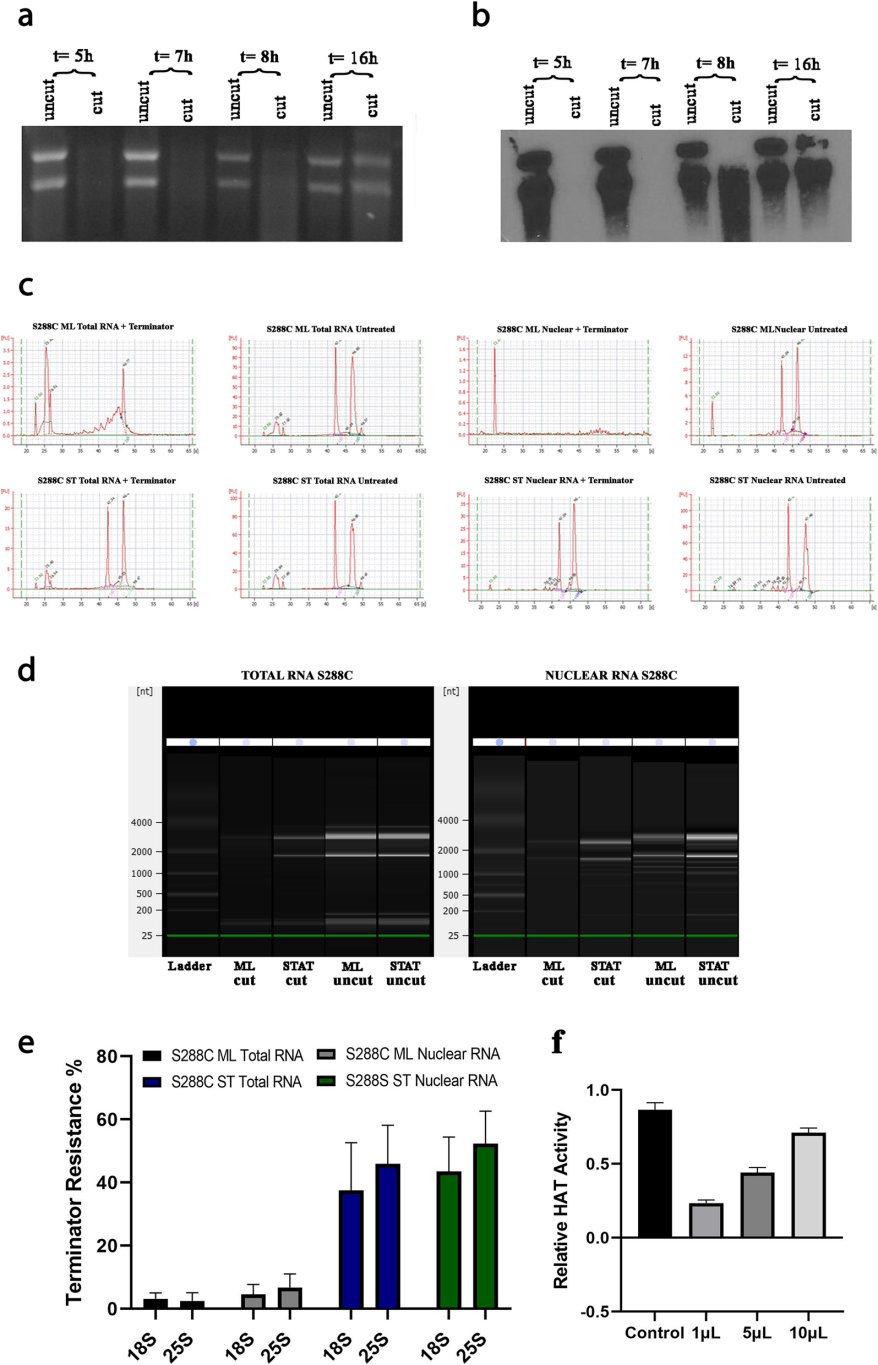
Terminator resistant 18S and 25S rRNA molecules in *Saccharomyces cerevisiae*. SYBR-gold-stained gel (**a**) and Northern blot (**b**) showing rRNA extracted at different time points either treated (cut) or untreated (uncut) by Terminator. (**c**) Electropherograms used to validate the quality of RNA and to confirm the presence of Terminator resistant molecules. (**d**) Gel image generated from electropherograms by bioanalyzer software. (**e**) Terminator resistance percentage of ribosomal and nuclear RNA extracted from mid log (ML) and stationary (ST) wild type *S. cerevisiae*. (**f**) Relative HAT activity of various amounts of *S. cerevisiae* nuclear extract. Error bars represent standard deviation from three different experiments. Gel and membrane were cropped to show relevant information. Full length gel and membrane with visible edges are shown in Fig. S1

Initially, we studied the behavior of wild type *Saccharomyces cerevisiae* regarding 18S and 25S rRNA resistance to Terminator digestion. As can be seen in Fig. 1, Terminator eliminates 18S and 25S rRNAs completely from total RNA isolated from wild type yeast cells during active growth period. As the cells approach the stationary growth phase, 18S and 25S rRNAs resistant to Terminator begin to appear. This can be seen directly in stained gels (Fig. 1a), as well as in Northern blotting (Fig. 1b). Furthermore, by assaying these molecules through a Bioanalyzer (Fig. 1c-d) we confirmed and quantitated 18S and 25S rRNAs resistant molecules. The area under the electropherogram peaks allowed us to quantitate them by comparing Terminator treated (cut) RNA to untreated (uncut) RNA (Fig. 1e). Figure 1f confirms that the origin of the nuclear RNA is indeed the nucleus. About 45% of the 18S and 25S RNAs become resistant to Terminator digestion during stationary growth phase. Terminator resistance in total RNA can result from either recapping in the cytoplasm or from de novo capping taking place in the nucleus. The fact that the percentage of Terminator resistance is similar or higher in nuclear RNA than it is in total RNA, indicates that a significant percentage of resistant molecules are produced in the nucleus and not in the cytoplasm. Hence the molecules are not recapped in the cytoplasm.

**Fig. 2:**
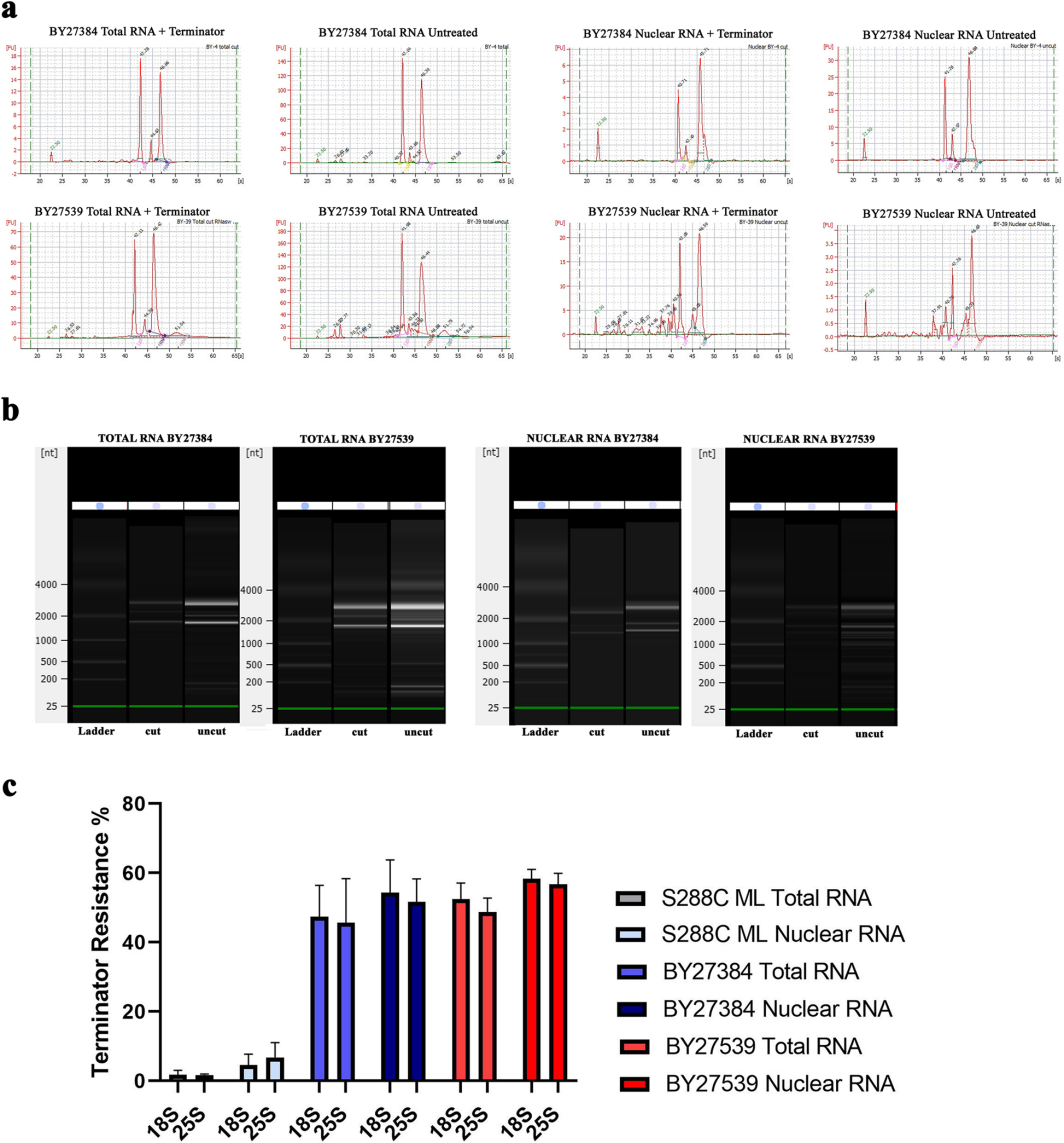
Terminator Resistance of rRNA in single (BY27384) and double mutant (BY27539) *S. cerevisiae*. (**a**) Electropherograms used to validate the quality of RNA and to confirm the presence of Terminator resistant molecules. (**b**) Gel image generated from electropherograms by bioanalyzer software. (**c**) Terminator resistance percentage of ribosomal and nuclear RNA extracted from mid log (ML), single mutant (BY27384) and double mutant (BY27539) *S. cerevisiae*. Error bars represent standard deviation from three different experiments

Figure 2 represents the Bioanalyzer assessment of ribosomal RNA from polymerase switched mutant *Saccharomyces cerevisiae.* As can be seen, both single and double mutant yeasts produce Terminator resistant 18S and 25S rRNA (Fig. 2a-c). This is true for both the total RNA and nuclear RNAs (Fig. 2c). In these mutants the percentage of resistant 18S and 25S components from nuclear extracts are similar or larger than those in total RNA, again indicating that some of the Terminator resistant molecules are produced in the nucleus and not modified in the cytoplasm.

**Fig. 3:**
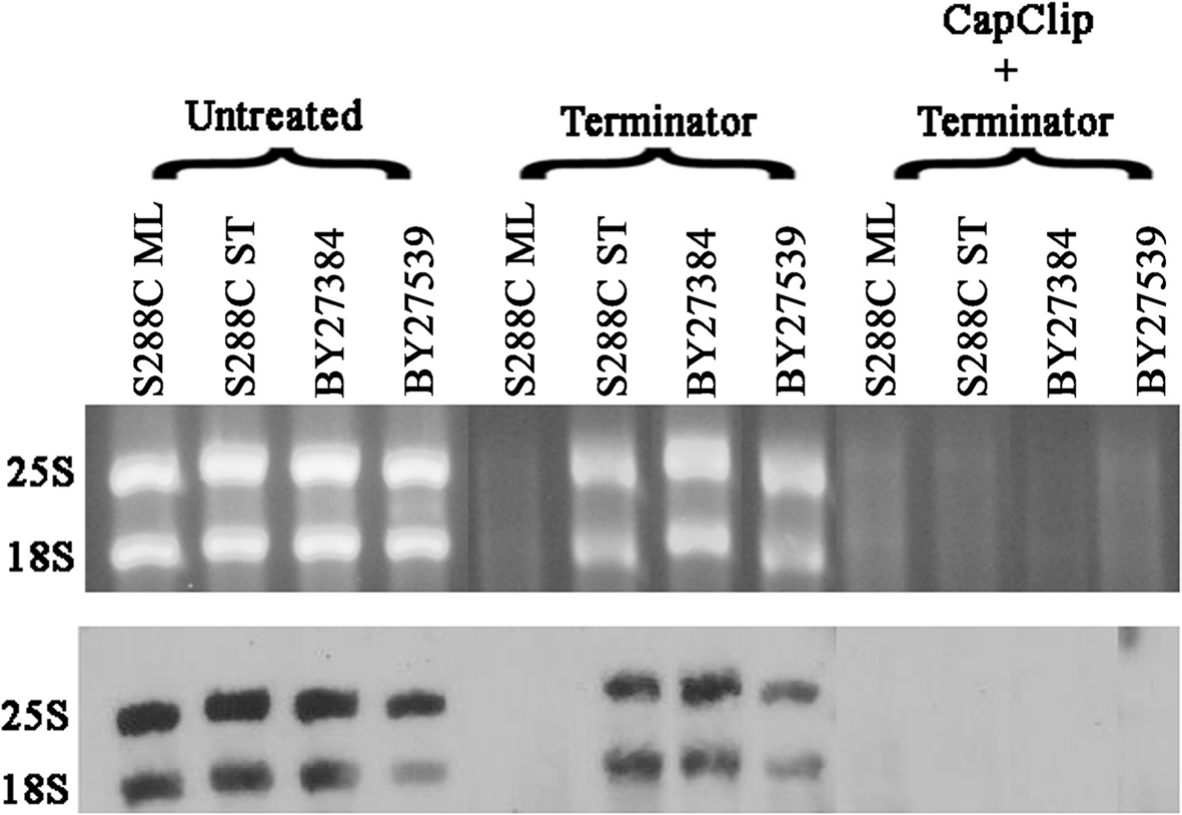
5’end analysis of 18S and 25S molecules in wild type and mutant *S. cerevisiae*. SYBR-gold-stained gel and Northern blot show the effect decapping followed by Terminator treatment on rRNA molecules 18S and 25S. Gel and membrane were cropped to show relevant information. Full length gel and membrane are shown in Fig. S2

Resistance to Terminator digestion of 18S and 25S rRNA can arise in several ways. The single phosphate usually present at the 5′-end of these processed molecules can be removed, resulting in a 5″-hydroxyl group or modified by addition to the single phosphate. This can include additional phosphate(s) with or without a cap structure or some other molecule. Data shown in Fig. 3 indicates that the addition of one or more phosphate is at least a part of the resistance. Terminator resistant rRNAs isolated from yeast in stationary phase or from the mutants are made susceptible to Terminator by first digesting them with a decapping enzyme (CapClip). These enzymes remove the cap structures from RNA by cutting between phosphates and can leave a single phosphate on the 5′-end of the RNAs making them susceptible to elimination by Terminator. This indicates, that at the minimum, Terminator resistant 18S and 25S molecules have more than one phosphate at their 5′-end.

**Fig. 4:**
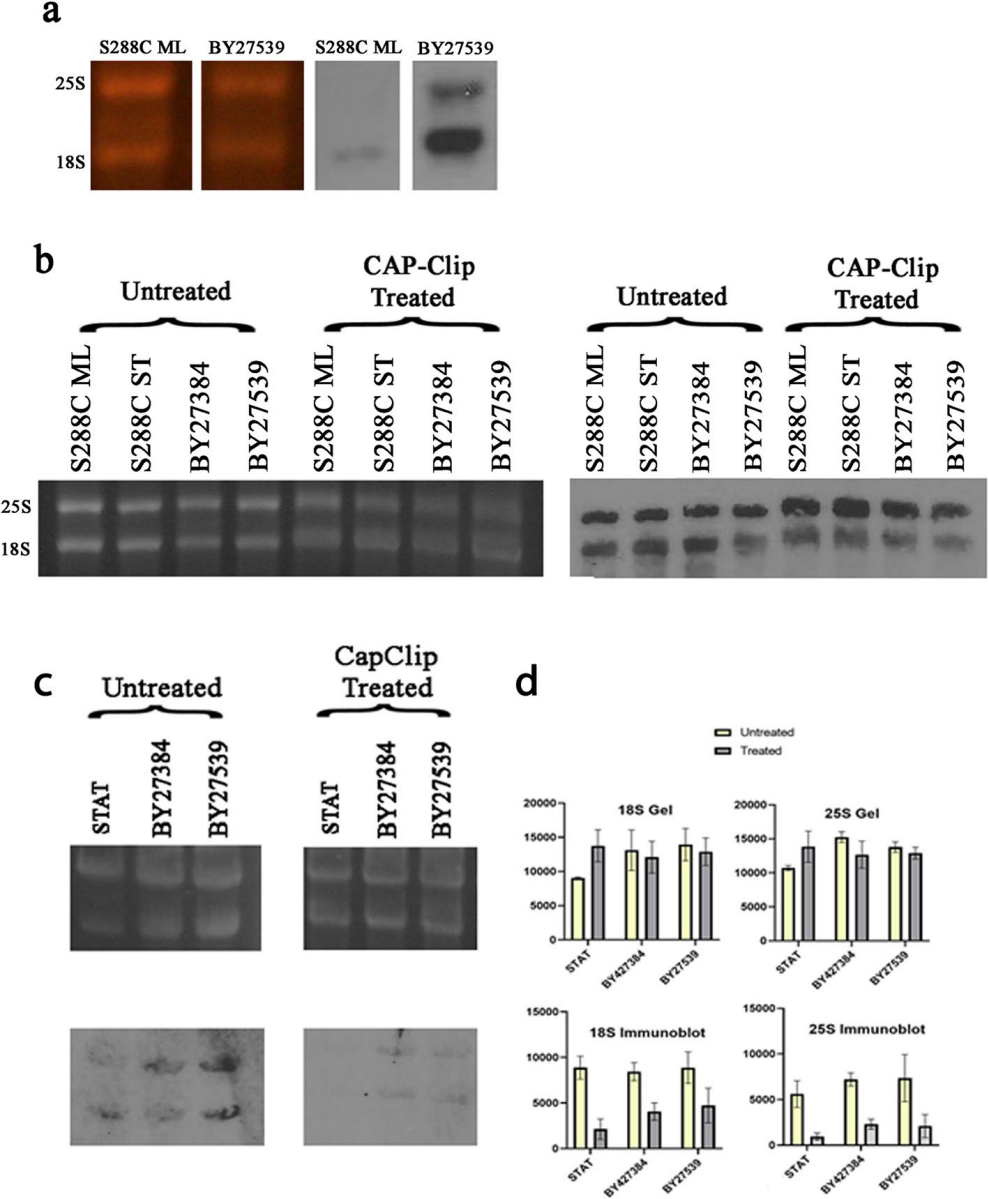
5′-cap analysis. (**a**) SYBR-gold stained gel and immunoblot using cap-specific antibody (H20) indicating presence of cap in mutant (BY2739) and wild type *S. cerevisiae* (S288C ML). (**b**) SYBR-gold stained gel and Northern blot with 18S and 25S probes showing rRNA that has been treated with CAP-Clip or untreated. Decapping enzyme does not degrade RNA. (**c**) Gel and corresponding immunoblot using H20 antibody. (**d**) Quantitation of gel and immunoblot bands using ImageJ software. Direct comparison of untreated and Cap-Clip treated RNA is shown in each graph. Mean and standard deviation were calculated from three different experiments. All the gels and membranes were cropped to show relevant information. Full length gels and membranes with visible edges are shown in Fig. S7

To see if any cap structure is present on these molecules, we utilized the trimethyl cap monoclonal antibody H20, widely used in cap detection studies [23]. As can be seen in Fig. 4a, the antibody strongly reacts with 18S and 25S rRNAs derived from the double mutant and minimally or not at all, with molecules isolated from wild type yeast in active growth phase. The weak signal from wild type yeast RNA may represent non-specific binding by the antibody. The stained gels show that the difference in intensity on the immunoblot is not related to differences in amount of RNA present. Decapping these molecules (Fig. 4c) decreases their immunoblot signals, again suggesting the presence of a cap on the phosphates. Both the gel and the Northern blot (Fig. 4b) show that the decapping enzyme does not degrade the RNA and therefore is not the reason for the decrease in immunoblot intensity. Fig. 4d represents the combined quantitative measurements of three experiments, one of them is shown in Fig. 4c and the other two are in Fig. S6.

## Discussion

The wild type *Saccharomyces cerevisiae* mimics the pattern of Terminator resistance we observed in *Candida albicans* [14]. These data are important for several reasons. First, during the active growth phase in the wild type cells, where RNAP I is well established to be the transcribing polymerase, we do not detect these Terminator resistant molecules. This makes a role for RNAP I in their production less likely. On the other hand, the fact that the double mutant, which has no functional RNAP I, only RNAP II for rRNA transcription, produces 18S and 25S rRNA at all, firmly establishes a role for RNAP II in the genesis of these molecules. The single mutant has a functional RNAP I but its efficiency in gaining access to its promoter is limited due to the UAF component mutation. To whatever extent it can access its promoter, it may produce some Terminator sensitive 18S and 25S and reduce the percentage of Terminator resistant 18S and 25S produced by the cell. This might explain why the single mutant produces them in smaller amounts as compared to the double mutant.

The mechanism underlying the production of Terminator resistant molecules, is unknown. It is well established that RNAP II can be involved in rRNA production in a polycistronic fashion in PSW yeast [16]. It has also been shown to gain access to rDNA promoter site during nutritional deprivation [17]. These polycistronic transcripts are processed into 18S and 25S components with a single phosphate at the 5′-end and therefore susceptible to be eliminated by Terminator. The fact that Terminator eliminates a percentage of 18S and 25S rRNA molecules clearly shows the existence of such molecules. What is unknown is how the rest of these RNA molecules produced by PSW yeast develop Terminator resistance.

Our data indicating that Terminator resistant 18S and 25S rRNA can be made Terminator sensitive by a de-capping enzyme (Fig. 3) establishes two things. First, that they have more than one phosphate at their 5′-end, as the de-capping enzyme is a pyrophosphatase, cutting exclusively between two phosphates; and second, that the molecules did not become Terminator resistant by having their single phosphate removed leaving them with a 5′-hydroxy end. The fact that these molecules are also detected by an anti-cap antibody and that decapping reduces the signal (Fig. 4), raises the possibility of a cap being attached to these extra 5′-phosphates. This would add another layer of protection against an exonuclease enzyme. Interestingly, polyadenylation, widely recognized as a function of RNAP II, has also been reported for rRNA 18S and 25S components in yeast [24–27] which fits well with our data. The observation where a transcribed RNAP II product gets modified by capping is not unusual, as it happens to all known RNA II transcripts, including mRNAs, miRNAs, lncRNAs, snoRNAs and snRNAs [28].

If indeed Terminator resistance in these molecules is related to extra phosphates with a possible cap attached then, the mechanism by which the cell achieves this is unknown. Our data is compatible with these molecules having tri-phosphates with a cap at their 5′-end. As all polymerases initiate transcription with nucleoside triphosphates (NTPs), in general an RNA molecule with three phosphates at its 5′-end can be newly transcribed, and if capped, it is by RNAP II. This is unlikely for these molecules, as there are no canonical sequences for an RNAP II associated promoter immediately upstream of either 18S or 25S.

The other modality would involve the modifying of processed 18S and 25S molecules initially transcribed in a polycistronic manner, similar to cytoplasmic recapping of decapped mRNAs [29]. The fact that RNAP II is involved in the formation of these molecules allows for some speculation. Capping of pre-mRNAs in *Saccharomyces cerevisiae*, is carried out co-transcriptionally by the capping enzyme complex (CE) made up of the triphosphatase Cet1 and the guanylyltransferase Ceg1 [30]. It is well established in yeast, that the capping enzyme complex interacts with the polymerase subunit of RNAP II at the C-terminal heptad repeats [31]. In fact, even the third enzyme involved with capping, namely N7 methyltransferase also interacts with the C-terminal repeats [32]. As processing of polycistronic rRNA can occur co-transcriptionally, if RNAP II is the transcribing polymerase of these 18S and 25S molecules, the capping machinery would also be available co-transcriptionally. The difficulty with this scenario is that our data shows that these molecules are produced in the nucleus. The Cet1 triphosphatase requires three phosphates as substrates at the 5′-position. Thus, there would have to be a kinase present in the nucleus capable in adding co-transcriptionally two phosphates to the single 5′-phosphate of the processed 18S and 25S molecules. There are examples that point to such a possibility. Cytoplasmic recapping of mRNAs has been shown in mammalian cells. Nudix family decapping enzymes such as DCP2, cleave between the α and β phosphates of the tri-phosphate cap leaving a 5′-monophosphate RNA in the cytoplasm. The nuclear mammalian capping complex RNGTT, is present in the cytoplasm also and for its triphosphates to function in recapping the 5′-monophosphate decapped mRNAs, they would need to have phosphates added to their 5′-end. Cytoplasmic capping enzyme complex with such kinase activity has been shown to be present in mammalian cells [29, 33]. Similarly, in the kinetoplastid *Trypanosoma brucei*, a cytoplasmic guanylyltransferase with 5′-RNA kinase activity capable of transforming a pRNA to a ppRNA, allowing a GMP transfer from GTP has been described [34].

In *Candida albicans* we have been able to detect Terminator resistant 18S and 25S rRNAs in ribosomes isolated from stationary yeast indicating that they are functional [14]. A potential for such degradation resistant molecules for the cell would be to maintain the protein producing capacity of the cell under nutritional duress. Our data indicates that this new role for RNAP II is not limited to mutational limitations of RNAP I but is present in wild type organisms during some part of the growth cycle, giving RNAP II an additional role in ribosomal production.

## Conclusions

Our findings of 5′-exonulease resistant 18S and 25S ribosomal molecules in polymerase switched *Saccharomyces cerevisiae,* confirms a role for RNAP II in the production of these molecules. This supports our previous published data regarding *Candida albicans,* where it is shown that RNAP II can produce such molecules in stationary growth phase cells, when the role of RNAP I has been downregulated. These findings point to another role for RNAP II in the production of ribosomes in addition to transcribing ribosome associated proteins.

## Methods

### Organisms

*Saccharomyces Cerevisiae* S288C (ATCC), BY27539 (*MAT****a*** *ade2–1 ura3–1 his3–11 trp1–1 leu2–3,112 can1–100 rrn9Δ::HIS3 rpa135Δ::LEU*) and BY27384 (*MAT****a****/a ade2–1/ade2–1 ura3–1/ura3–1 his3–11/his-3-11 trp1–1/trp1–1 leu2–3,112/leu2–3,112 can1–100/can1–100 RRN9/rrn9Δ::HIS3*) (YGRC/NBRP Japan) were maintained in 50% glycerol in YPD broth (2% w/V tryptone, 1% w/v yeast extract, 2% w/v dextrose) at − 80 °C. Cells were activated in YPD broth at 30 °C and maintained on Sabouraud dextrose agar at 4 °C, passaged every 4–6 weeks up to 4–5 times. Yeasts were lifted from agar surface and grown in YPD broth for variable length of times at 30 °C. Yeast cell concentrations were established using a hemocytometer.

### RNA isolation

Cells were collected by centrifugation, washed with sterile phosphate buffered saline (PBS) and were put on ice pending total RNA extraction. Cells were disrupted with RNase-free zirconia beads and RNA was isolated using Ambion RiboPure RNA Purification kit for yeast (Ambion/ThermoFisher, AM1926)) according to the manufacturer’s instructions.

Nuclear RNA was obtained using the Yeast Nuclei Isolation kit (Abcam, ab206997) following the manufacturer’s instructions. Histone Acetyltransferase (HAT) Activity Assay Kit (Abcam, ab65352) was used to verify nuclear source of isolated RNA (Fig. 1f). RNA quantification and quality were assessed by using a Qubit 4 fluorometer and an Agilent 2100 Bioanalyzer.

### Terminator 5′-phosphate-dependent exonuclease experiments and 5′-end analysis

Total RNA was treated with Terminator (Lucigen, TER51020) following the manufacturer’s protocol using the supplied Buffer A. The ratio of enzyme to substrate employed was 1 U per 1 μg of RNA to ensure adequate cleavage. RNase inhibitors (NEB) were used in all the assays at a 1 μg/ml concentration.

### RNA analysis

Terminator treated and non-treated RNA samples were loaded into an RNA 6000 Nano chip and analyzed with the Agilent 2100 bioanalyzer system (Agilent Technologies, INC). Electropherograms were used to calculate Terminator resistance percentages by measuring the areas under the peaks of untreated (uncut) RNA and dividing it by the area of treated (cut) RNA.

### Northern blotting and immunoblotting

RNA was separated on formaldehyde agarose gels (Lonza) and stained with SYBR Gold Nucleic Acid Gel Stain (Life Technologies) for 30 min. Gel images were captured with a digital camera (Canon Vixia HFS30). RNA was transferred by electro-blotting (BIO-RAD Trans-Blot Turbo Transfer System) to a positively charged nylon membrane (Millipore) in 0.5 x TBE (standard Tris/Borate/EDTA buffer). The RNA was cross-linked to the membrane using UV (Stratagene UV Crosslinker). For immunoblotting, the membrane was blocked with 10% Block Ace™ (Bio-Rad) for 30 min at 25 °C, followed by the addition of anti-m3G-cap, m7G-cap antibody clone H20 (Millipore Sigma) diluted 1:1000 in 10% Block ACE™ and incubated for 24 h at 4 °C. Goat anti-mouse conjugated to HRP was added to the membrane at 1:5000 in blocking solution for 30 min at 25 °C. The Prosignal™ (Prometheus) chemiluminescence substrate was used to detect the HRP signal. Film was developed with the SRX-101A Konica film processor. For Northern blotting, we used the North2South Chemiluminescent Hybridization and Detection Kit (ThemoFisher, 17,097) following the manufacturer’s protocol. Probes specific for 25S and 18S components of the ribosomal RNA were prepared by PCR using specific 18S and 25S primers. PCR products were cloned into TOPO® pcr4 vector followed by transformation with TOP-10 chemically competent cells. Several colonies were screened for insert presence and sequenced (Laragen Inc). For probe preparation, bacteria were grown in Terrific Broth (Fisher) with ampicillin at 50 μg/ml, and plasmids were isolated with a QuickLyse Kit (Qiagen). Inserts were released with BamH I/EcoR I for 25S and BamH I/NcoI for 18S, and were purified with a QIAquick Gel Extraction Kit (Qiagen). 50–100 ng of purified) inserts were biotinylated using the EZ-Link™ Psoralen-PEG3-Biotin (ThermoFisher).

### Gel and immunoblot analysis

Band quantitation of scanned images was performed by open-source ImageJ software (https://imagej.nih.gov/ij/index.html). Each image was processed to have the same resolution. Area and pixel densities were measured from three different experiments after converting the image to gray-scale. All the images that were used to generate these results are shown in Fig. S6.

### Decapping assays

Cap-Clip™ acid pyrophosphatase (Cellscript) was used according to manufacturer instructions for decapping RNA samples. Verification of cap removal was done by gel electrophoresis, Northern blotting and immunoblotting using anti-cap (H20) antibody. Biotinylated probes used for Northern blot were made using the following sequences: 18S_3_Fwd (5′-GTGAAACTCC GTCGTGCTGGG-3′), 18S_3_Rev (5′-TAATGATCCTTCCGCAGGTTCAC CTAC-3′), 25S_3_Fwd (5′-AACGCGGTGATTTCTTTGCTCCAC-3′), 25S_3_Rev (5′-GGCTTAATCT CAGCAGATCGTAACAACAAGG-3′).

## Supplementary Information

**Additional file 1: Fig S1** Full-sized images of the SYBR-gold stained gel and Northern blot shown in Fig. 1. Lanes 1=uncut 5hr; 2=cut 5hr; 3=uncut 7hr; 4=cut 7hr; 5=uncut 8hr; 6=cut 8hr; 7=uncut 16hr; 8=cut 16hr. The selected area indicates the lanes depicted in Fig. 1. **Fig S2** Full-sized images of the SYBR-gold stained gel and Northern blot shown in Fig. 3. Lanes 1=S288C ML untreated; 2=S288C ST untreated; 3=BY27384 untreated; 4=BY27539 untreated; 5= S288C ML Terminator treated; 6=S288C ML CapClip + Terminator; 7=S288C ST Terminator treated; 8=S288C ST CapClip + Terminator; 9=BY27384 CapClip + Terminator; 10=BY27384 Terminator treated; 11=BY27539 CapClip + Terminator; 12=BY27539 Terminator treated. The selected areas indicate the lanes depicted in Fig. 3. Lanes were cropped and rearranged in Fig. 3 to organize each treated group. **Fig S3.** Full-sized images of the SYBR-gold stained gel and Immunoblot shown in Fig. 4a. Lanes 1=S288C ML 2μg; 2=S288C ML 1μg; 3=S288C ML 0.5μg; 4=BY27539 2μg; 5=BY27539 1μg; 6=BY27539 0.5μg. The selected areas were used in Fig. 4a. **Fig. S4.** Full-sized images of the SYBR-gold stained gel and Northern blot shown in Fig. 4b. Lanes 1=S288C ML untreated; 2=S288C ST untreated; 3=BY27384 untreated; 4=BY27539 untreated; 5=S288C ML Cap-clip treated; 6=S288C ST CapClip treated; 7=BY27384 CapClip treated; 8=BY27539 CapClip treated. The selected areas were used in Fig. 4b. **Fig S5.** Full-sized images of the SYBR-gold stained gel and Immunoblot shown in Fig. 4c. Lanes 1=S288C ML CapClip treated; 2=S288C ST CapClip treated; 3=BY27384 CapClip treated; 4=BY27539 CapClip treated; 5=S288C ML untreated; 6=S288C ST untreated; 7=BY27384 untreated; 8=BY27539 untreated. The selected areas indicate the lanes depicted in Fig. 4c. Lanes were cropped and rearranged in Fig 4c. **Fig S6.** Images of scanned gels and immunoblots that were used to quantitate Terminator resistance before and after Cap-Clip treatment. Area and density of each band was measured using ImageJ software. Results were obtained by determining the area under each band peak (immunoblots) or in between peaks (gels) from three different experiments and are depicted in Fig. 4d. **Fig S7** Images of full blots and membranes with visible edges that were cropped in **Fig S6** and were used to calculate band areas with ImageJ software.

## Data Availability

All data generated and/or analyzed during the study are included in this published article (and its supplementary information files).
