# Supplementary material for: RNAP II produces capped 18S and 25S ribosomal RNAs resistant to 5′-monophosphate dependent processive 5′ to 3′ exonuclease in polymerase switched Saccharomyces cerevisiae
Source: BMC Mol Cell Biol. 2022 Apr 10;23:17. doi: 10.1186/s12860-022-00417-6 (PMC8994892; doi:10.1186/s12860-022-00417-6)
Supplement: Supplementary file 1 — Additional file 1: Fig S1 Full-sized images of the SYBR-gold stained gel and Northern blot shown in Fig. 1. Lanes 1=uncut 5hr; 2=cut 5hr; 3=uncut 7hr; 4=cut 7hr; 5=uncut 8hr; 6=cut 8hr; 7=uncut 16hr; 8=cut 16hr. The selected area indicates the lanes depicted in Fig. 1. Fig S2 Full-sized images of the SYBR-gold stained gel and Northern blot shown in Fig. 3. Lanes 1=S288C ML untreated; 2=S288C ST untreated; 3=BY27384 untreated; 4=BY27539 untreated; 5= S288C ML Terminator treated; 6=S288C ML CapClip + Terminator; 7=S288C ST Terminator treated; 8=S288C ST CapClip + Terminator; 9=BY27384 CapClip + Terminator; 10=BY27384 Terminator treated; 11=BY27539 CapClip + Terminator; 12=BY27539 Terminator treated. The selected areas indicate the lanes depicted in Fig. 3. Lanes were cropped and rearranged in Fig. 3 to organize each treated group. Fig S3. Full-sized images of the SYBR-gold stained gel and Immunoblot shown in Fig. 4a. Lanes 1=S288C ML 2μg; 2=S288C ML 1μg; 3=S288C ML 0.5μg; 4=BY27539 2μg; 5=BY27539 1μg; 6=BY27539 0.5μg. The selected areas were used in Fig. 4a. Fig. S4. Full-sized images of the SYBR-gold stained gel and Northern blot shown in Fig. 4b. Lanes 1=S288C ML untreated; 2=S288C ST untreated; 3=BY27384 untreated; 4=BY27539 untreated; 5=S288C ML Cap-clip treated; 6=S288C ST CapClip treated; 7=BY27384 CapClip treated; 8=BY27539 CapClip treated. The selected areas were used in Fig. 4b. Fig S5. Full-sized images of the SYBR-gold stained gel and Immunoblot shown in Fig. 4c. Lanes 1=S288C ML CapClip treated; 2=S288C ST CapClip treated; 3=BY27384 CapClip treated; 4=BY27539 CapClip treated; 5=S288C ML untreated; 6=S288C ST untreated; 7=BY27384 untreated; 8=BY27539 untreated. The selected areas indicate the lanes depicted in Fig. 4c. Lanes were cropped and rearranged in Fig 4c. Fig S6. Images of scanned gels and immunoblots that were used to quantitate Terminator resistance before and after Cap-Clip treatment. Area and density of each band was measured using ImageJ softwa [file 12860_2022_417_MOESM1_ESM.docx]

**Fig S1** Full-sized images of the SYBR-gold stained gel and Northern blot shown inf Figure 1. Lanes 1=uncut 5hr; 2=cut 5hr; 3=uncut 7hr; 4=cut 7hr; 5=uncut 8hr; 6=cut 8hr; 7=uncut 16hr; 8=cut 16hr. The selected area indicates the lanes depicted in Figure 1.


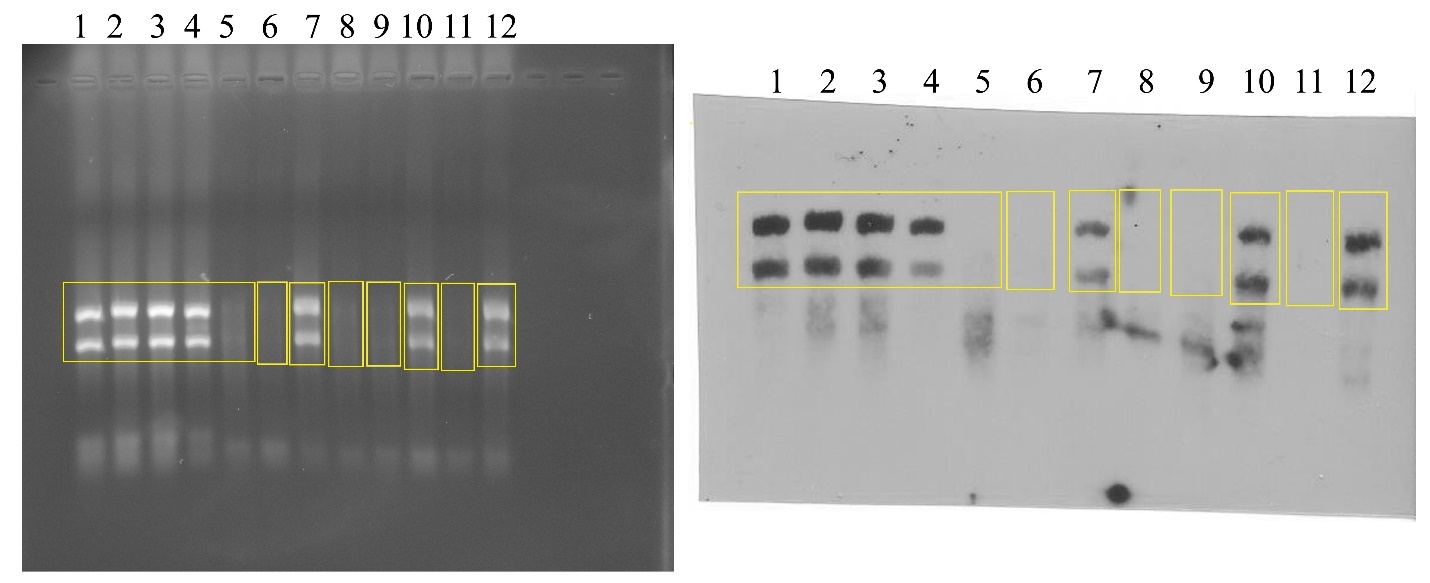


**Fig S2** Full-sized images of the SYBR-gold stained gel and Northern blot shown in Figure 3. Lanes 1=S288C ML untreated; 2=S288C ST untreated; 3=BY27384 untreated; 4=BY27539 untreated; 5= S288C ML Terminator treated; 6=S288C ML CapClip + Terminator; 7=S288C ST Terminator treated; 8=S288C ST CapClip + Terminator; 9=BY27384 CapClip + Terminator; 10=BY27384 Terminator treated; 11=BY27539 CapClip + Terminator; 12=BY27539 Terminator treated. The selected areas indicate the lanes depicted in Figure 3. Lanes were cropped and rearranged in Figure 3 to organize each treated group.


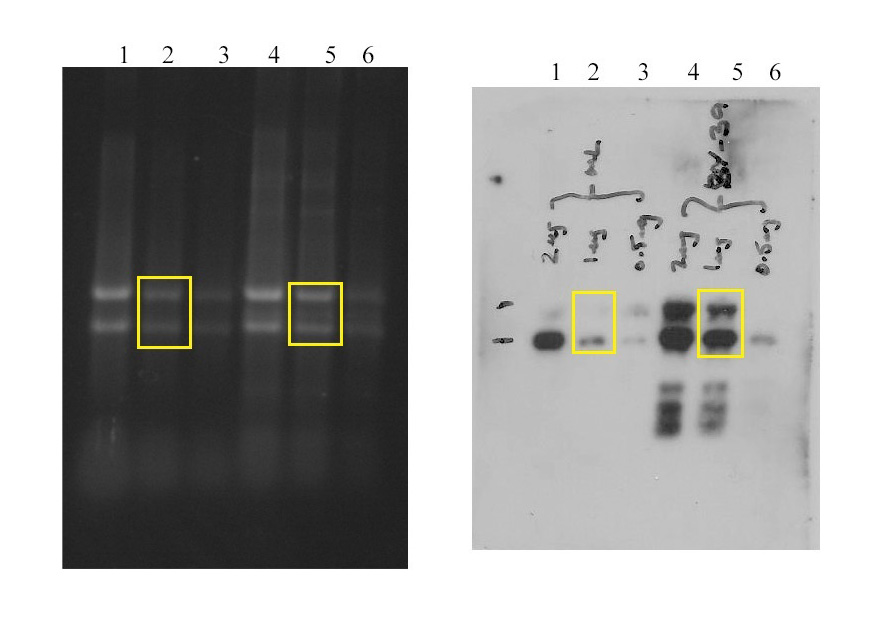


**Fig S3**. Full-sized images of the SYBR-gold stained gel and Immunoblot shown in Figure 4a. Lanes 1=S288C ML 2µg; 2=S288C ML 1µg; 3=S288C ML 0.5µg; 4=BY27539 2µg; 5=BY27539 1µg; 6=BY27539 0.5µg. The selected areas were used in Figure 4a.


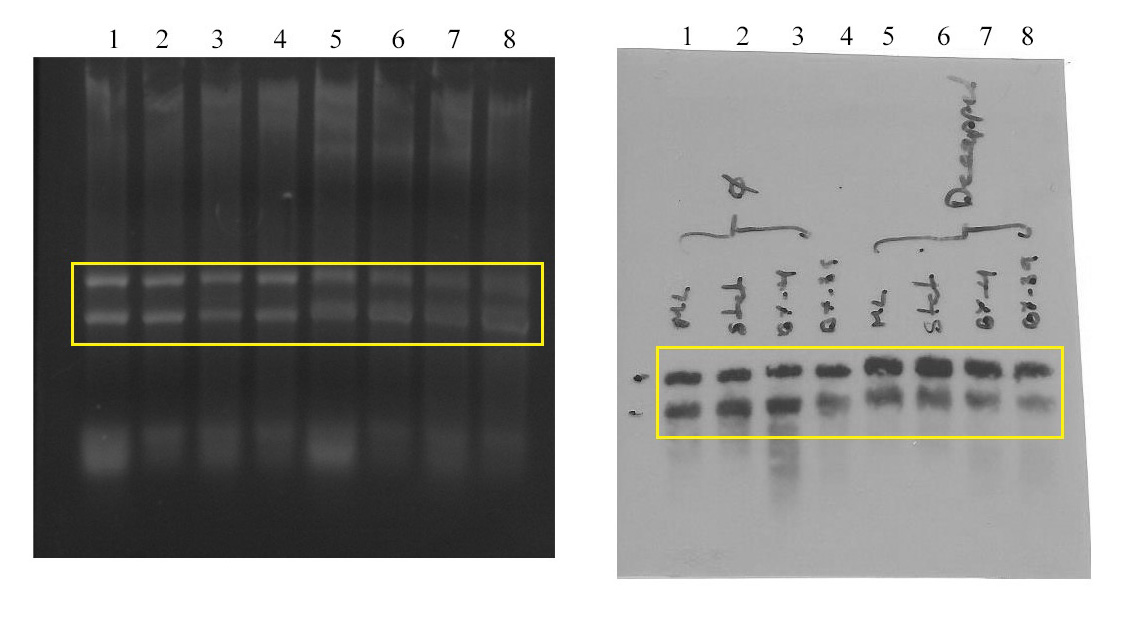


**Fig. S4**. Full-sized images of the SYBR-gold stained gel and Northern blot shown in Figure 4b. Lanes 1=S288C ML untreated; 2=S288C ST untreated; 3=BY27384 untreated; 4=BY27539 untreated; 5=S288C ML Cap-clip treated; 6=S288C ST CapClip treated; 7=BY27384 CapClip treated; 8=BY27539 CapClip treated. The selected areas were used in Figure 4b.


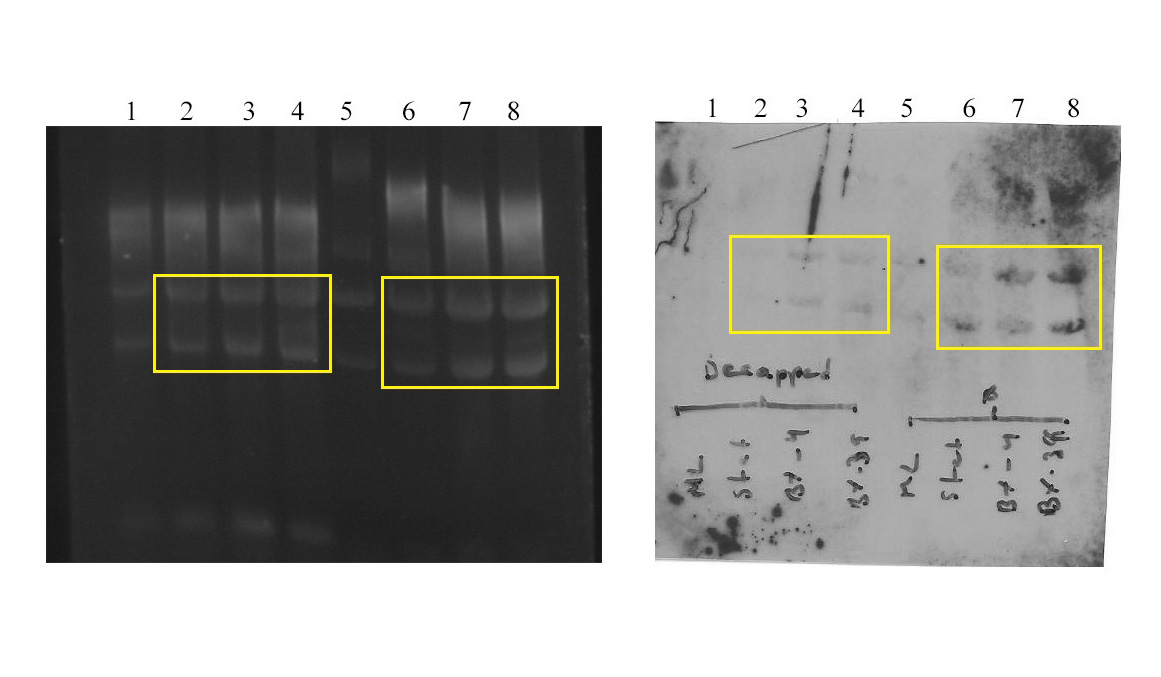


**Fig S5**. Full-sized images of the SYBR-gold stained gel and Immunoblot shown in Figure 4c. Lanes 1=S288C ML CapClip treated; 2=S288C ST CapClip treated; 3=BY27384 CapClip treated; 4=BY27539 CapClip treated; 5=S288C ML untreated; 6=S288C ST untreated; 7=BY27384 untreated; 8=BY27539 untreated. The selected areas indicate the lanes depicted in Figure 4c. Lanes were cropped and rearranged in Fig 4c.

**Fig S6**. Images of scanned gels and immunoblots that were used to quantitate Terminator resistance before and after Cap-Clip treatment. Area and density of each band was measured using ImageJ software. Results were obtained by determining the area under each band peak (immunoblots) or in between peaks (gels) from three different experiments and are depicted in Figure 4d.


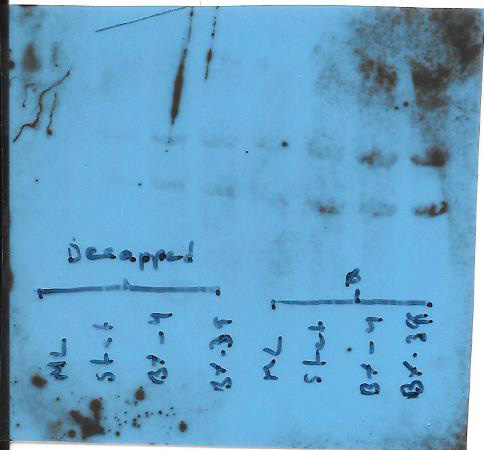

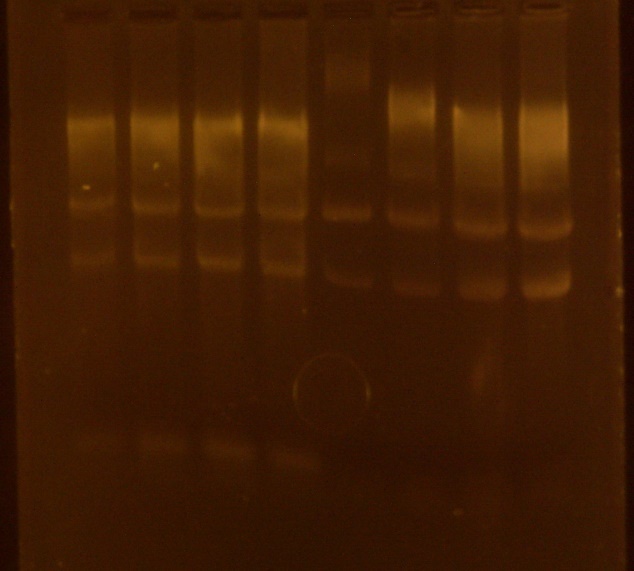


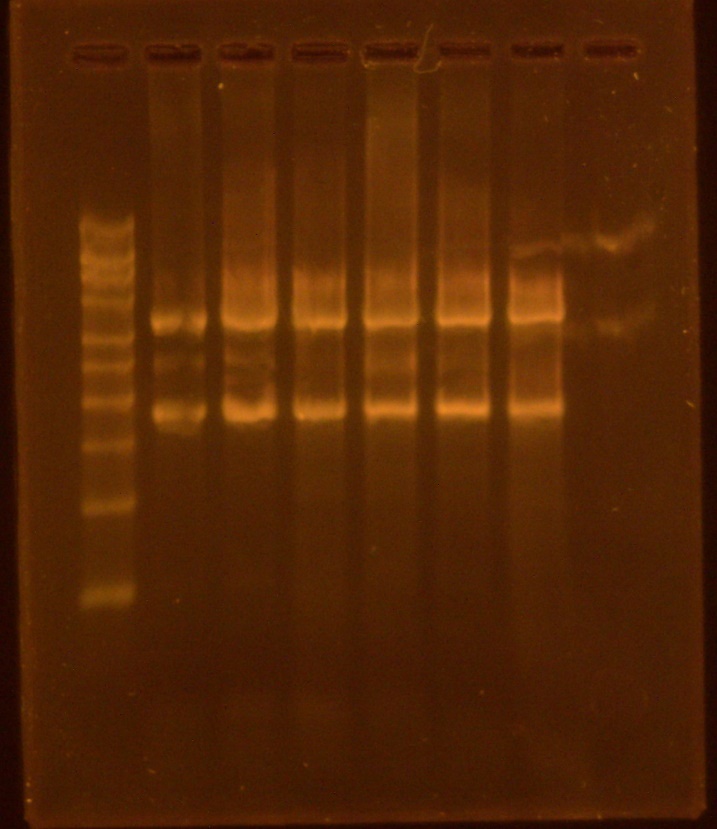


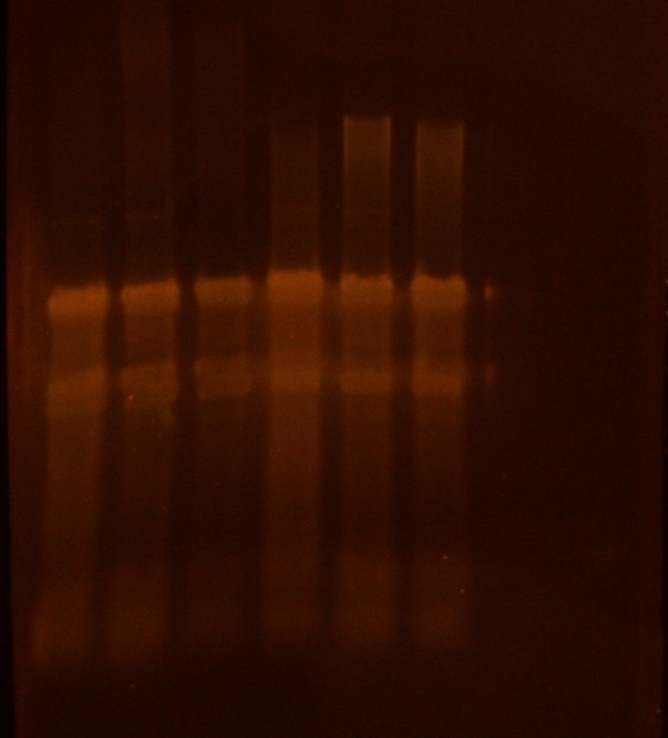


**Fig S7** Images of full blots and membranes with visible edges that were cropped in Fig S6 and were used to calculate band areas with ImageJ software.
